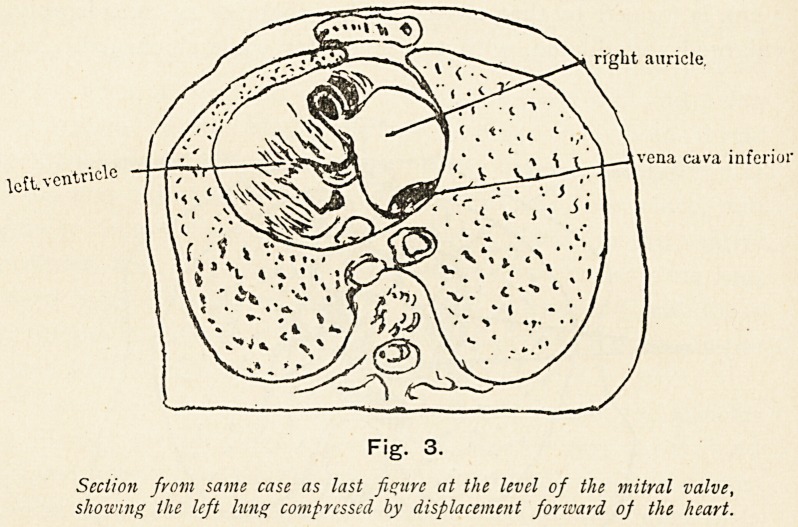# The Influence of the Auricles on the Percussion of the Heart

**Published:** 1903-06

**Authors:** D. R. Paterson

**Affiliations:** Senior Assistant-Physician, Cardiff Infirmary; Hon. Physician, Seamen's Hospital, Cardiff; Lecturer on Materia Medica and Pharmacology, Cardiff Medical School.


### THE INFLUENCE OF THE AURICLES ON THE PERCUSSION OF THE HEART.

BY

D. R. PATERSON, M.D., M.R.C.P.,

*Senior Assistant-Physician, Cardiff Infirmary; Hon. Physician, Seamen's Hospital, Cardiff; Lecturer on Materia Medica and Pharmacology, Cardiff Medical School.*

---

THE influence of the auricles upon the position of the heart has hardly received from clinicians the attention its importance demands; and though the work [1] of Dr. Ewart, dealing with the recognition of enlargements of the left auricle by posterior percussion, has stimulated interest in this practical question, something still remains to be done. We are, indeed, familiar with the description of the morbid anatomy of the auricles in various diseases of the heart; but the part they play in modifying its percussion boundaries has been generally lost sight of. Perhaps the reason for this lies in the usual method of conducting a *post-mortem* examination, where opening of the chest involves disturbance of the relations of the organs.

No systematic attempt has apparently been made to ascertain the effect of enlargement of one auricle upon the other, or upon the heart as a whole; and it was with a view to obtain some information on this point that I carried out, some time ago, a large series of frozen sections in dogs, where the parts could be examined *in situ* without disturbance. A later opportunity was afforded me, through the kindness of my colleague, Professor Dixon, of examining a case of mitral disease in the frozen human subject by means of sections; and the result of both investigations, which I desire to record here, go to show that the auricles play a not unimportant part in modifying the relations of the heart.

In a paper published some time ago, on the use of posture in percussion of the cardiac area,[2] I showed that the organ was possessed of considerable mobility. The force of gravity,

[1] *Brit. M. J.*, 1899, ii. 1167. [2] *Ibid.*, 1898, i. 135.

acting in change of posture, brings about an alteration in its position. Thus, when the body is placed in the prone position the heart falls forward, and a larger area of its surface comes into contact with the anterior chest wall; and it is clear that the organ has not only a side-to-side movement, but one also in the antero-posterior plane. That it is considerable is proved by the action of such a minor force as gravity, and it is obvious that more powerfully-acting causes within the organ itself will produce an even greater result. During my investigations on the influence of gravity on the position of the heart, the effect of the dorsal posture of the animal sometimes differed in an interesting way. Instead of falling back towards the vertebral column, and its anterior surface becoming enveloped by the lungs, as is usual, the heart was sometimes found well forward, in contact with the front wall of the chest, a relationship associated with the prone position. The condition which interfered in this way with the effect of gravity was the distension of the auricular cavities, which had carried the anterior surface of the heart forward; and when this was prominent, it sufficed to bring a well-contracted ventricle close under the chest wall, as if death had taken place in the prone position.

The anatomical position of the left auricle has been the subject of some curious misconceptions. Some of the older writers, among whom was Walshe, asserted that it came into relation with the anterior chest wall, and might be revealed by pulsation in the third intercostal space on the left side. Dr. G. W. Balfour is still of opinion that pulsation in that site may be auricular, and finds support in it for his view that the systolic bruit heard in the pulmonary area in anæmia is due to regurgitation through the mitral orifice, and conducted to the surface by the dilated auricle. The erroneousness of this contention has been demonstrated, and it is now recognised that the auricle is placed deeply in the chest, though there still appears to be confusion in the minds of some recent writers, who seem disposed to believe, with Walshe, that "direct physical evidence of dilation of either auricle is only to be had by percussion [anteriorly] in the natural sites of those cavities."[1]

[1] *Diseases of the Heart*, 4th Edition, 1873, p. 325.

For a clear understanding of the effect of the auricle in displacing the heart, it will be necessary to allude to a few anatomical details.

The left auricle may be described as an expanded tube, formed by the junction of the right and left pulmonary veins, which enter from the root of the lung on either side. It is situate almost in the middle line, in front of the sixth, seventh, and eighth dorsal vertebræ, and is placed almost entirely behind the heart, forming the back part of that organ. It forms, according to Cunningham, a greater portion of the posterior wall of the auricular portion of the heart than the right auricle, the latter cavity, indeed, coming, to some extent, to the front, in connection with the right side of the base. The site of the left auricle is well brought out by an illustration in Spalteholz's work,[1] which shows it forming the posterior wall of the heart, with the pulmonary vein entering on either side. It is frequently assumed that the auricles are placed on either side of the heart, as indicated by their names; but the partition between the two auricles—septum atriorum,—instead of lying in an antero-posterior direction, forms the posterior wall of the right auricle; so that this cavity might as correctly be termed the anterior auricle, as it occupies a plane in front of the other which might not inaptly be named the posterior. This is well shown by one of Braune's sections, of which I reproduce a tracing. (Fig. 1.)

On the posterior wall of the left auricle the pulmonary veins open; and as these structures take part in the formation of the root of the lung, it is obvious that the back part of the auricle is the most fixed, and that any enlargement of it must necessarily take place in a forward direction. The posterior wall comes into close apposition to the œsophagus in front of the spinal column, a relationship which has made it possible for tracings of the auricle to be obtained by means of an air tampon passed down the gullet as far as the base of the heart. As a distended auricle can only expand in an anterior direction, it follows that the heart as a whole will be thrust forward in the same plane, and, as a result, the organ will be moved closer to the anterior chest-wall.

[1] *Handatlas der Anatomie.*

Fig. 1.

*Sagittal section of the chest, showing the right auricle lying in front of the left auricle (after Braune).*

In the movements of the heart observed in the open chest, the left auricle becomes swollen during systole, pushing the base of the ventricle before it as it comes forwards and descends, or, as it is expressed by Sibson, "the left auricle, being filled from behind forwards, lifts up and tilts forward the left ventricle at its auricular attachment." This movement forward will be greatly increased by an abnormal distension of the auricle, and will affect the whole heart rather than a portion. As it lies not only behind the left ventricle, but is also posterior to the right auricle, it will carry the right side of the heart forward as well; and the right auricle being frequently enlarged under the same conditions, the movement of the organ will be considerable, and will express itself by the anterior aspect flattening against the chest wall. Distension of both auricles will therefore contribute very materially to the increase of the cardiac dulness, and make it possible for them to considerably alter the percussion boundaries. It is usually assumed that extension of the cardiac dulness means general enlargement of the organ; but I think it is clear, both from the effects of posture and distension of the auricles, that alteration of the percussion area may take place without any great enlargement of the heart, and mainly as a result of displacement.

Some time ago I investigated this question by means of frozen sections of the dog's chest. The animals, placed in various positions before being killed, were after death frozen, and sections made without disturbance of the parts. A large series of such sections went to prove that distension of the auricles with the animal in the dorsal position was sufficient, in some instances, to counteract the effect of gravity, and instead of the front of the heart receding from the chest wall and being overlapped by the lungs, the whole organ had been thrust forwards close under the ribs. With little or no auricular distension this forward displacement did not take place, and gravity came into play. In one animal, placed in the ventral position, the flattening of the cardiac surface was marked, aided largely by dilated auricles, whilst in a second, with these chambers small and contracted, the organ failed to reach the chest wall, and was enveloped by the lungs.

The value of ascertaining the exact relations of the auricles in disease of the mitral valve is, of course, very great; and I have been fortunate enough to have recently had an opportunity of examining, by the section method, the body of a man who had suffered from marked mitral obstruction and regurgitation. The cadaver was embedded in a freezing mixture, and transverse sections were made by my colleague, Professor Dixon. An examination of the heart showed the cusps of the mitral valve stiff and thickened, and the auriculo-ventricular ring hard and calcareous. The left auricle was dilated, and its wall hypertrophied, the right corresponding chamber being also much distended. There was a moderate degree of hypertrophy of the left ventricle, and the organs generally showed signs of chronic venous congestion.

One of the sections passing through the base of the heart exposed the highest point of the left auricle, whilst the section immediately below that cut through the lower part of the same chamber, intersecting the mitral valve, so that a close examination of the relations of the auricle was possible. In Fig. 2, which is a photographic tracing of the upper section seen from above, the posterior situation of the auricle is brought out. It forms at this level the back wall of the heart, in front of the œsophagus and spinal column, whilst anterior to it lie the origins of the great vessels and the right auricle. This section measured 9 cm. transversely at the widest part, which was the left auricle, and antero-posteriorly the axis is 8 cm., of which 3 cm. is formed by that cavity. This chamber is considerably enlarged and dilated, with the muscular walls thickened. Its

Fig. 2.

*Transverse section of human chest from a case of mitral stenosis, passing through the base of the heart and opening at two points the left auricle, the outline of which is represented by the interrupted line.*

interior is roughly pyramidal in form, the posterior wall being fixed and the anterior shelving forwards; so that the greatest depth of the cavity from before backwards is at the lower part, where it passes into the left ventricle through the mitral valve. From these relations I think it clear that any enlargement of the auricle must take place in a forward and downward direction.

In Fig. 3, in which the section passes through the mitral valve, the transverse diameter of the organ measures 12 cm., of which the greatly-dilated right auricle forms 5½ cm. The heart is placed almost wholly to the left of the middle line, a position largely due, it will be observed, to the distension of the right auricle. It is likewise in close apposition to the chest wall, and part of the left lung bears an interesting relation to it. This is markedly compressed, and flattened into a thin, tongue-like process, which is fleshy and devoid of air. This result is

Fig. 3.

*Section from same case as last figure at the level of the mitral valve, showing the left lung compressed by displacement forward of the heart.*

explained by the existence of pleural adhesion, which has prevented recession of the lung before the advancing heart; and the compact, firm appearance of the pulmonary tissue is evidence of considerable forward pressure.

In the section below this, taken a little above the apex, the heart is in close contact with the chest wall, and flattened; and no part of the lung intervenes over the whole area.

I think it clear, from a study of these sections, that dilatation of the left auricle carries forward the base of the heart, and moves the whole organ downwards and forwards; that the right auricle assists this movement, and contributes a displacement to the left as well; whilst the general result is, to bring the heart into closer apposition to the chest wall and increase the area which may be revealed by percussion.

If we seek evidence from the experimental side, we shall find that the series of changes observed in artificial stenosis in the open chest gives support to the same contention. When a ligature is placed around the aorta and gradually tightened, the heart enlarges, and advances towards the front wall of the chest. It is true that all the cavities increase in size; but the antero-posterior displacement is too great to be explained merely by ventricular enlargement, and there is no difficulty in determining that engorgement of the left auricle is an important factor in this movement. When the aorta is freed there is a marked recession of the organ, along with the shrinking of the auricles.

One of the most striking attributes of the heart is its power of adapting itself to changed conditions. Dilatation of the cavities occurs in purely physiological circumstances; that is to say, where the residual blood in the chambers of the heart at the termination of systole is above normal in amount. This physiological dilatation does not necessarily mean heart failure: it occurs frequently, and is quite recoverable from, within certain limits. When it is marked the heart will, of course, be pushed forward and flattened on the chest wall, presenting a larger surface to percussion, and giving the usual signs of increased cardiac area. These signs, usually associated with general pathological enlargements of the heart, may be brought out in some degree by dilatation of the auricles alone. Any relief to the auricles, by diminishing the residual blood and the consequent dilatation, will lessen this forward pressure and produce a recession of the heart, and the assumption of the round and more restricted shape.

From what has already been said, it is very probable that a comparatively small diminution of the auricle is sufficient to alter the percussion in a pronounced manner, inasmuch as the falling back of the organ will be followed by a return of the lungs on the anterior aspect of the heart.

This fact will, I think, serve to explain, in some degree, the alteration in the physical signs of the heart in cases that have undergone resistance exercises and baths. These two procedures will assist in emptying the auricles of their residual blood, and relieve the dilatation. Some effect will be exercised upon the size of the ventricles as well; but it is exceedingly probable that a not unimportant factor in the diminution of cardiac dulness, about which so much has been written, is the falling backwards of the heart, consequent on the relief of the distended auricles. It must be borne in mind that it does not require an extensive movement forward to materially increase the cardiac dulness; and it is equally true that a moderate relief of the auricles will result in diminution of the percussion limits.

Cohnheim has pointed out that in all cases of cardiac insufficiency, be the original defect or the cause of the failure of compensation what it may, there recurs a dilatation of one or more of the cavities. In mitral incompetence the systole drives blood back into the auricle, and this cavity becomes abnormally distended: as a result, there is increased diastolic dilatation; and where there is, in addition, stenosis of the valve, the amount of residual blood in the auricles is great enough to cause displacement of the heart, as the sections from the human cadaver show. According to Guttmann,[1] where stenosis of the left auriculo-ventricular orifice reaches a certain degree of intensity the apex-beat is absent, whilst the impulse of the base is much increased in force and spreads over a larger surface than naturally. This statement I have repeatedly confirmed by clinical observation; and the explanation is, I think, that urged in this paper—viz., the effect of the distended auricles.

In conclusion, I should like to point out that my investigations go to show that, whilst enlargement of the left auricle may take place to some extent downwards, and possibly slightly upwards, the chief increase is undoubtedly in an antero-posterior direction. On the other hand, Dr. William Ewart contends that in mitral stenosis percussion may determine an increase in the postcordial dulness, from enlargement of the auricle. If my observations are correct, this can take place only to a limited degree; for it is clear, from what has been already said, that any distension of that chamber will carry the heart forward, and rather tend to influence the precordial area of dulness.

[1] *Physical Diagnosis*, 1879, p. 214.

## Figures and Tables

**Fig. 1. f1:**
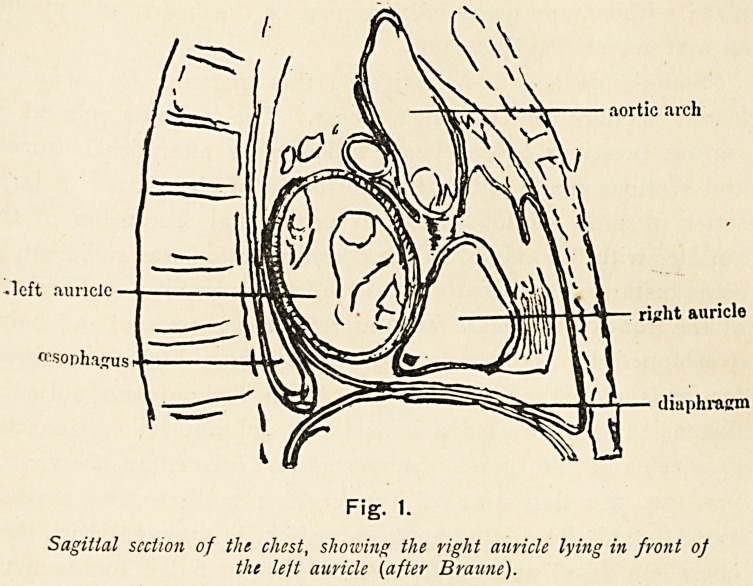


**Fig. 2. f2:**
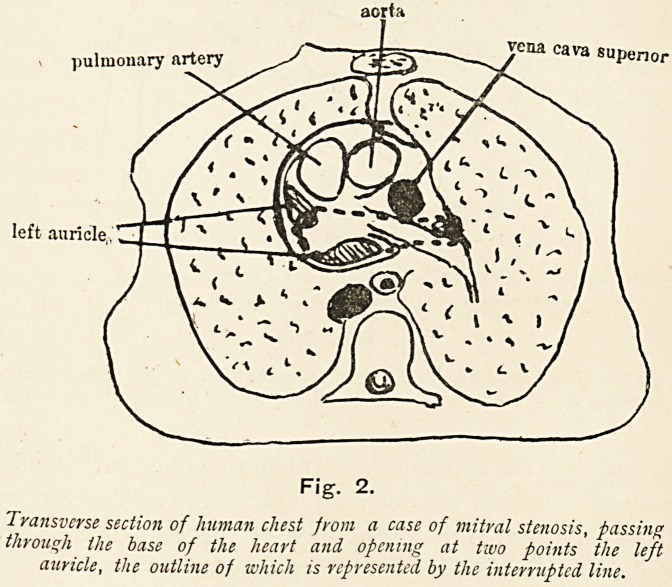


**Fig. 3. f3:**